# Effects of S24–7 on the weight of progeny rats after exposure to ceftriaxone sodium during pregnancy

**DOI:** 10.1186/s12866-021-02231-0

**Published:** 2021-06-03

**Authors:** Xin Yang, Ting Tang, Jing Wen, Mengchun Li, Jie Chen, Tingyu Li, Ying Dai, Qian Cheng

**Affiliations:** 1 Department of Primary Child Health Care, Children’s Hospital of Chongqing Medical University, Chongqing, 400014 China; 2 National Clinical Research Center for Child Health and Disorders, Ministry of Education Key Laboratory of Child Development and Disorders, Chongqing, China; 3 Chongqing Key Laboratory of Child Health and Nutrition, Chongqing, China

**Keywords:** Ceftriaxone sodium, S24–7, Valine, Leucine and isoleucine biosynthesis

## Abstract

Antibiotic exposure during pregnancy will adversely affect the growth of offspring; however, this remains controversial and the mechanism is poorly understood. To study this phenomenon, we added ceftriaxone sodium to the drinking water of pregnant rats and continuously monitored the body weight of their offspring. The results showed that compared with the control group, the offspring exposed to antibiotics during pregnancy had a higher body weight up to 3 weeks old but had a lower body weight at 6 weeks old. To determine the role of the gut microbiota and its metabolites in the growth of offspring, we collected feces for sequencing and further established that the experimental group has a different composition ratio of dominant bacteria at 6 week old, among which S24–7 correlated negatively with body weight and the metabolites that correlated with body weight-related unique flora were L-Valine, L-Leucine, Glutaric acid, N-Acetyl-L-glutamate, and 5-Methylcytosine. To further explore how they affect the growth of offspring, we submitted these data to Kyoto Encyclopedia of Genes and Genomes website for relevant pathway analysis. The results showed that compared with the control, the following metabolic pathways changed significantly: Valine, leucine, and isoleucine biosynthesis; Protein digestion and absorption; and Mineral absorption. Therefore, we believe that our findings support the conclusion that ceftriaxone sodium exposure in pregnancy has a long-lasting adverse effect on the growth of offspring because of an imbalance of gut microbiota, especially S24–7, via different metabolic pathways.

## Introduction

In recent years many diseases during pregnancy have been treated using antibiotics [12]; however, the risk of antibiotic exposure during pregnancy is gradually increasing [5]. Researchers have found that the growth of neonates is different from between those treated with and without antibiotics, and there is increasing, but controversial, evidence that exposure to antibiotics during pregnancy might have both short- and long-term effects on babies.

A recent study [1] showed that prenatal exposure to antibiotics reduced the birth weight of babies by approximately 138 g; however it has been found that antibiotic exposure during pregnancy was associated with the incidence of childhood obesity [24]. After multivariate adjustment, a meta-analysis did not observe any association between antibiotic use during pregnancy and overweight in school-age children [26]. Similarly, studies also confirmed that antibiotic use does not affect the risk of small for gestational age (SGA) or large for gestational age (LGA) birth weight in pregnant women [16]. In addition, population-based studies [8, 9] have shown that prenatal exposure to antibiotics is not associated with overweight offspring. These studies have reported opposite results about the causal relationship between repeated exposure to antibiotics and body weight [17]; therefore, the effect of antibiotic exposure on offspring during pregnancy still needs further research.

The effects of antibiotics depends on the gut microbiome [6], which can promote growth and maintain the normal physiological activities of the human body via its metabolites. It has been found [10] that antibiotics can treat necrotizing enterocolitis (NEC) by changing the structure of the gut microbiome, which affected the abundance of 44 metabolites in different metabolic pathways in animal models of premature infants, including tryptophan metabolism and kynurenine pathway. It has been [28] observed that antibiotic-induced gut microbiome disorders in rats, disturbed glycine, serine, and threonine metabolism; niacin and nicotinamide metabolism; and bile acid metabolism; and metabolic pathways related to antibiotic-induced gut microbiome disorders showed obvious changes.

Therefore, we hypothesized that antibiotic exposure during pregnancy is likely to have an important effect on offspring and this effect might even persist, which might be caused by an imbalance of the gut microbiome and changes in related metabolic pathways. However, the specific mechanism still needs further research. This study aimed to clarify whether antibiotic exposure during pregnancy plays a role in the effects of the gut microbiota on the offspring and affect their weight gain.

## Experimental procedures

### Experimental animals

Specific Pathogen Free (SPF) grade female and male Sprague-Dawley rats were purchased from the Experimental Animal Center of Chongqing Medical University and kept at the Animal Center of Children’s Hospital of Chongqing Medical University [under experimental license [SCXK(Yu) 2012–0015]. All animal experimental procedures were performed in line with the ethical standards set by the ethics committee of the Animal Center of Children’s Hospital of Chongqing Medical University, was also carried out in compliance with the ARRIVE guidelines.

The male and female rats were caged overnight at 1:1, and the female rats whose vaginal plugs were found in the cage the next morning were recorded as the first day of pregnancy (Gestational day 01, GD01), and were kept separately. The pregnant rats were randomly divided into two groups: The control pregnant group (*n* = 6) did not receive any treatment in their drinking water; the anti-pregnant group (*n* = 6) received ceftriaxone sodium (concentration 1 mg/ml, daily intake is the normal adult dose) [2, 13, 15] in their drinking water during GD05 to GD11 of pregnancy. Litters were standardised to 8–9 pups within 24 h of birth (4 males and 4 females, where possible), the ratio of female to male in each litter of the two group was approximately 1:1 and without statistical difference. Finally, the offspring in the control group were named as Control group (*n* = 55), and offspring in the antibiotic group were named as the Antibiotic group (*n* = 50). Offspring were measured the body weight at Post-natal day 3 (PND3), 1st week, 2nd week, 3rd week and 6th week, and were randomly selected in each litter for inspection at 2nd week, 3rd week and 6th week. When the experiment is over, put the rats in a closed container and slowly infuse carbon dioxide gas for about 5–10 min until lose life.

### Monitoring of weight

To investigate the effects of antibiotic exposure during pregnancy on the offspring, we added ceftriaxone sodium to the drinking water of rats and continuously monitored the body weight of the offspring after birth. Post-natal day 3 (PND3), 1st week, 2nd week, 3rd week and 6th week were used to measure the body weight of each group.

### Gut microbiota sequencing

To determine whether the antibiotic had an effect on the pregnant rats and to study the role of the gut microbiota in the growth of the offspring, we collected feces for sequencing. The feces at GD05 and GD12 of the pregnant rats and the feces of the pups at 2, 3_,_ and 6 weeks old were collected and sequenced by Shanghai Personal Biotechnology (Shanghai, China). The experimental steps followed the manufacturer’s instructions.

1. Microbial total DNA extraction: The DNA was quantified using an ultraviolet spectrophotometer after extraction of total DNA.
2. Target fragment PCR amplification: According to the conserved regions in the target sequence, the corresponding primers were designed incorporating sample-specific barcode sequences, and then the rRNA gene variable region or a specific gene fragment was subjected to PCR amplification.
3. Amplification and purification of the amplified product: The PCR amplification product was detected using 2% agarose gel electrophoresis, and the target fragment was subjected to gel recovery using an AXYGEN (Union city, CA, USA) kit.
4. Fluorescence quantification of the amplification products: With reference to the preliminary quantitative results of electrophoresis, and the quantification of the PCR amplification products, the products were quantified using the fluorescent reagent in the Quant-iT PicoGreen dsDNA Assay Kit using a Microplate reader (BioTek, Winooski, VT, USA; FLx800).
5. Sequencing library preparation: Sequencing libraries were prepared using Illumina’s TruSeq Nano DNA LT Library Prep Kit (Illumina, San Diego, CA, USA).
6. High-throughput sequencing was performed using a MiSeq sequencer with the corresponding MiSeq Reagent Kit V3 (600 cycles).

### Metabolomics test

To determine the differences in metabolites between offspring exposed to antibiotics during pregnancy and Control, we collected the feces of the two groups at 6 weeks old and performed non-targeted metabolomics analysis based on ultra-high performance liquid chromatography (UHPLC), which was carried out by Shanghai Personal Biotechnology.

After metabolite extraction, we used liquid chromatography–tandem mass spectrometry (LC-MS/MS) analysis to identify and quantify the metabolites. The samples were separated using an Agilent 1290 Infinity LC Ultra High Pressure Liquid Chromatograph (Agilent)(Waters, ACQUITY UPLC BEH Amide 1.7 μm, 2.1 mm × 100 mm column). The quadrupole time of flight (QTOF) mass spectrometry conditions comprised: Electrospray ionization (ESI) positive and negative ion modes for detection, separation of the samples using UHPLC, and mass spectrometry performed using a Triple TOF 6600 mass spectrometer (AB SCIEX, Framingham, MA USA).

Data processing: The raw data was converted to the .mzXML format using ProteoWizard, and then peak aligned using the XCMS program. Retention time correction and extraction of the peak area were performed. Metabolite structure identification using accurate mass matching (< 25 ppm) and secondary, The method of spectral matching comprised retrieving the self-built database of the laboratory.

### Statistical analysis

All statistical operations were performed using GraphPad Prism 7.0 statistical software (GraphPad Software, Inc., La Jolla, CA, USA). The measurement data were expressed as the mean ± standard error (x ± SEM). The mean value comparison between the two groups was assed using a t test, the rate comparison was performed using a chi-squared test, and the weights were evaluated using analysis of variance with repeated measurement data. *P* < 0.05 indicated that the difference is statistically significant.

## Results

### Microflora structure of the feces of pregnant rats

To verify whether the structure of the microbiota in pregnant rat feces changed after antibiotic exposure, we collected the feces and performed DNA sequencing. To screen the differences in the members of the microbiota, multivariate statistical analysis was performed in accordance with the relative abundance of each of the bacteria in the sample to obtain the variable importance of projection (VIP) score. Differentially abundant bacteria were identified using a VIP > 1 and an independent sample t test *P* < 0.05.

**Fig. 1:**
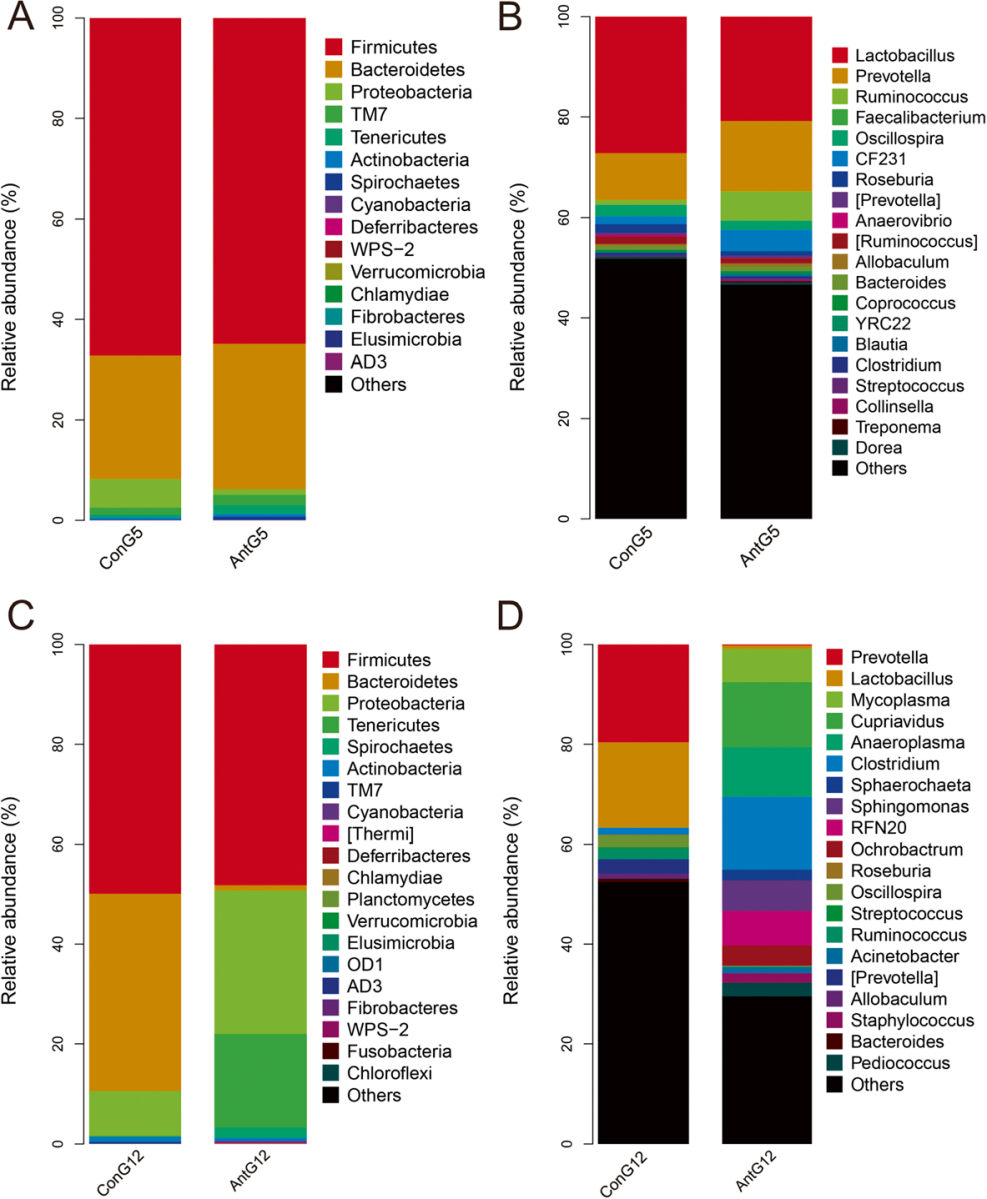
The gut microbiota was detected in the feces of pregnant rats. **A** Gut microbiota sequencing was performed for two groups at gestational day (G)5 at the phylum level. **B** Gut microbiota sequencing was performed for two groups at G5 at the genus level. **C** Gut microbiota sequencing was performed for two groups at G12 at the phylum level. **D** Gut microbiota sequencing was performed for two groups at G12 at the genus level. (n _Control pregnant rat group_ = 6, n _anti-pregnant rat group_ = 6)

In the taxonomic composition analysis at the phylum level, the dominant bacteria in the Control at GD05 were mainly Firmicutes (67.2%), Bacteroidetes (24.5%), and Proteobacteria (5.7%), and the antibiotic group they were Firmicutes (58.9%), Bacteroidetes (36.3%), and Proteobacteria (1.4%)(Fig. 1A). At the genus level, the dominant bacteria in the Control at GD05 were mainly Lactobacillus (27.1%), Clostridiales (15.4%), S24–7 (11.7%), and Prevotella (9.4%), and in the antibiotic group they were Lactobacillus (18.8%), Prevotella (27.3%), and Clostridiales (7%) (Fig. 1B).

The dominant bacteria in the Control at GD12 were mainly Firmicutes (49.9%), Bacteroidetes (39.5%), and Proteobacteria (9%) at the phylum level, and in the antibiotic group they were Firmicutes (48.2%), Bacteroidetes (1%), Proteobacteria (28.8%), and Tenericutes (18.7%) (Fig. 1C). At the genus level, the dominant bacteria in the Control at GD12 were mainly Prevotella (19.6%), Lactobacillus (17.1%), S24–7 (11.1%), and Clostridiales (9.1%), and in the antibiotic group they were Prevotella (1.8%), Lactobacillus (15.6%), Cupriavidus (12.9%), Clostridiales (14.6%)(Fig. 1D). These results showed that the gut microbiota of pregnant rats changed significantly after exposure to antibiotics.

### Effect of antibiotic exposure during pregnancy on the weight of offspring

To investigate whether antibiotic exposure during pregnancy affects offspring, we added ceftriaxone sodium to the drinking water of pregnant rats and continuously monitored the body weight of their offspring after birth.

**Fig. 2:**
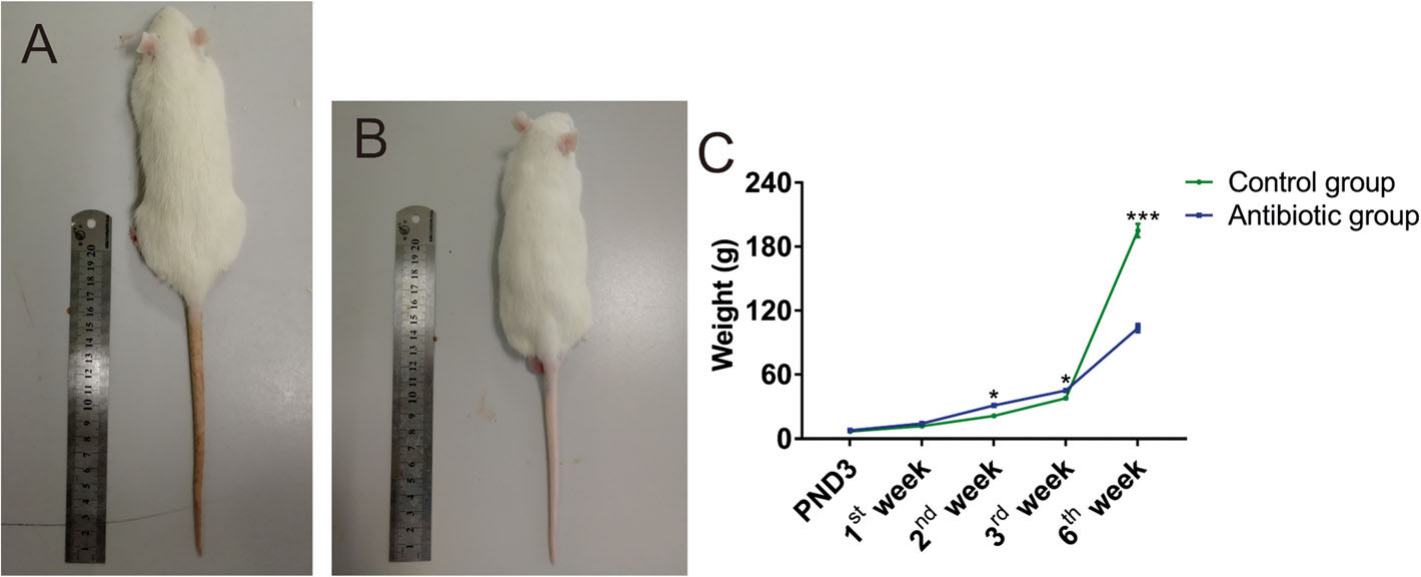
The weight gain trend of the offspring in the two groups of rats. **A** The image of a rat in the Control at age of 6 weeks old. **B** The image of a rat in the antibiotic group at age of 6 weeks old. **C** There was no significant difference in the weight from birth to 1 week old between the two groups, the offspring exposed to antibiotics during pregnancy (C, *n* = 50) had a higher body weight than the Control (C, *n* = 55) at 2 and 3 weeks old, but had a lower weight at age of 6 weeks old (A, n _Control group_ = 30; B, n _antibiotic group_ = 26) .*P < 0.05, **P < 0.01, ***P < 0.001

The weight of each group of young rats was measured dynamically after birth and the results of continuous testing for 6 weeks are shown in Fig. 2C. There was no significant difference in the weight of the offspring at birth between the two groups (Fig. 2C, *P* > 0.05). Compared with the Control group, the offspring exposed to antibiotics during pregnancy had a higher body weight at 2 and 3 weeks old (Fig. 2C, *P* < 0.05), while their weight was significantly lower than that of the Control at 6 weeks old (Fig. 2C, *P* < 0.001). The offspring in the antibiotic group were smaller than the Control group at 6 weeks old(Fig. 2A, B).

### Differences in gut microbiota between the offspring

To further study the role of gut microbiota in the growth of offspring, we collected their feces for sequencing of bacterial DNA.

**Fig. 3:**
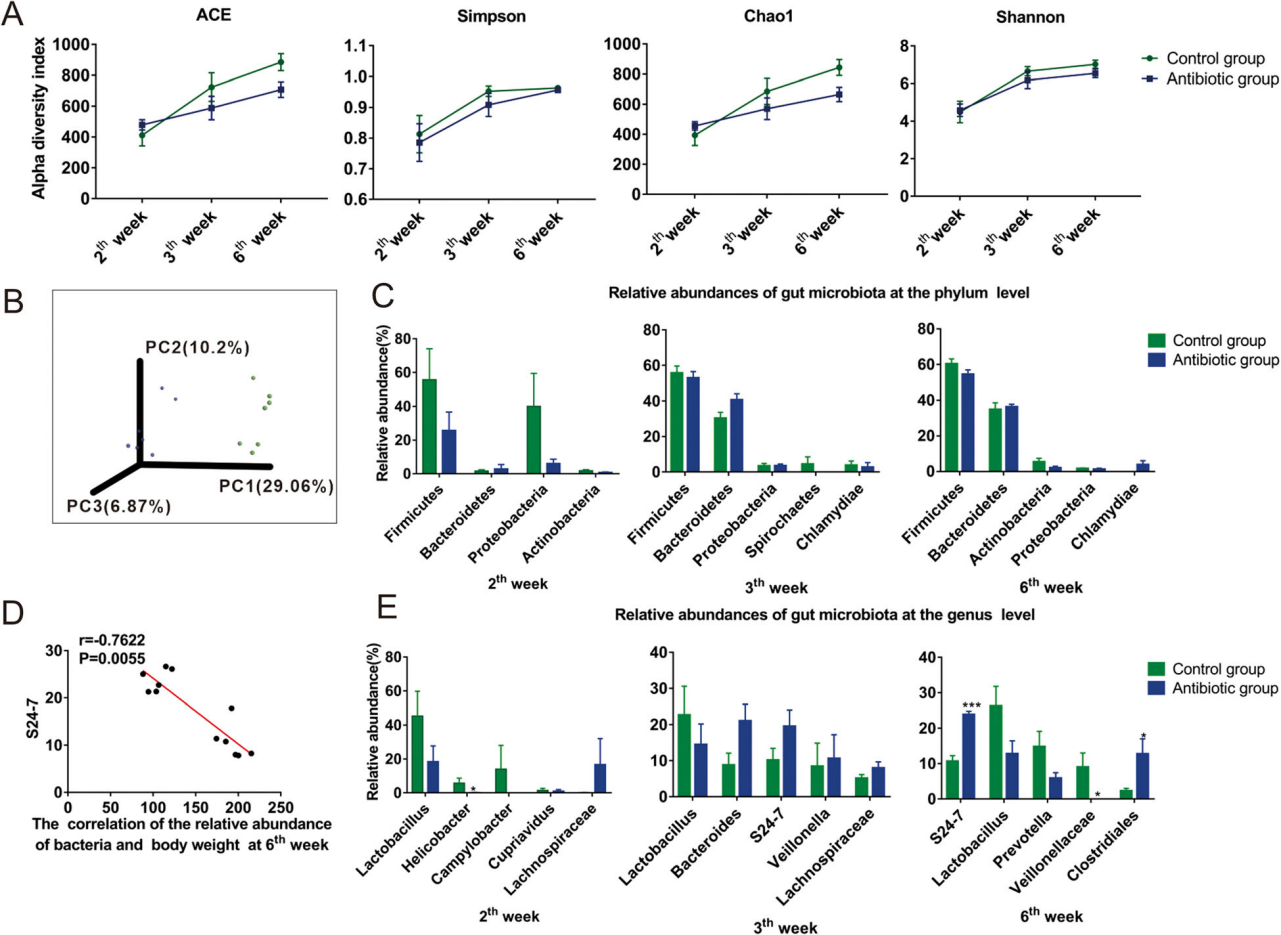
The gut microbiota was detected in the feces of two groups. **A** α diversity analysis results. **B** β diversity analysis results. **C** Gut microbiota sequencing was performed for the two groups at the phylum level. **D** Pearson correlation analysis between differential metabolites and body weight. **E** Gut microbiota sequencing was performed for the two groups at the genus level. **F** The relative abundance of S24–7 in the two groups.(n _Control group 2nd week_ = 4, n _Control group 3rd week_ = 6, n _Control group 6th week_ = 7, n _antibiotic group 2nd week_ = 6, n _antibiotic group 3rd week_ = 6, n _antibiotic group 6th week_ = 7). *P < 0.05, **P < 0.01, ***P < 0.001

Alpha diversity analysis was used to analyze the complexity of the gut microbial community. Compared with the control group, the offspring exposed to antibiotics during pregnancy had lower indexes (Chao1 and ACE, which represent the abundance of gut microbiota, Shannon index and Simpson index, which represent the diversity of gut microbiota); however, the differences were not statistically significant(Fig. 3A, *P* > 0.05). Beta diversity analysis was used to compare and analyze the microbial community structure between the different samples. Principal coordinate analysis (PCoA) showed that the samples of the experimental group and the control were far apart, indicating that the composition of the gut microbiota is significantly different between them (Fig. 3B).

In the taxonomic composition analysis at the phylum level the dominant bacteria in the Control at 3 week old were mainly Firmicutes (56.9%), Bacteroidetes (30.7%), Spirochaetes (3.7%), and Proteobacteria (3.6%), and in the antibiotic group they were Firmicutes (54.8%), Bacteroidetes (39%), Proteobacteria (3.5%), and Chlamydiae (2.6%). At 6 weeks old, the dominant bacteria in the Control were mainly Firmicutes (58.7%), Bacteroidetes (31.8%), and Actinobacteria (4.7%), and in the antibiotic group they were Firmicutes (56.1%), Bacteroidetes (34.7%), and Chlamydiae (3.3%). The composition and proportion of predominant bacterial groups in the antibiotic group at 2 weeks old were different from those of the Control, however, at 6 weeks there was no statistical difference (Fig. 3C).

At the genus level, the dominant bacteria in the Control at 3 weeks old were mainly Lactobacillus (19%), Veillonella (14.3%), S24–7 (11.4%), Bacteroides (9.3%), and Prevotella (5.6%), and in the antibiotic group they were Lactobacillus (25.9%), Bacteroides (20%), S24–7 (16.8%), Lachnospiraceae (7%), and Enterococcus (5.9%). The dominant bacteria in the Control at 6 weeks old were mainly Lactobacillus (23.3%), Prevotella (14%), S24–7 (10%), and Veillonellaceae (7.9%), while in the antibiotic group they were S24–7 (23.3%), Lactobacillus (11.5%), Clostridiales (11.2%), and Phascolarctobacterium (8.1%) (Fig. 3E). At the genus level, the composition and proportion of predominant bacteria in the antibiotic group and Control group were different, and continued from 2 weeks old to 6 weeks old. There was a statistically significant difference in the relative abundance of S24–7 (Fig. 3E, *P* < 0.001), Clostridiales (Fig. 3E, *P* < 0.05), and Veillonellaceae (Fig. 3E, *P* < 0.05) between the antibiotic and Control groups.

To further study the relationship between the differential gut microbiota and weight, we conducted a correlation analysis and found that the abundance of S24–7 has a significant negative correlation with weight (Fig. 3D, *P* < 0.001). We then compared the abundance of S24–7 in the control group and the antibiotic group respectively, and found that the relative abundance of S24–7 3 and 6 weeks old was significantly higher compared with that at 2 weeks old in both groups (Fig. 3F, *P* < 0.05). In addition, the relative abundance of S24–7 in the antibiotic group was higher than that of the control at all time points, and the difference was significant at 6 weeks old (Fig. 3F, *P <* 0.05).

### Differences in metabolites between the two offspring groups

To determine whether the metabolites in the two groups of offspring were different, we collected the feces of the two groups at 6 weeks old and performed non-targeted metabolomics analysis based on UHPLC and performed multidimensional statistical analysis using Orthogonal partial least squares discriminant analysis (OPLS-DA). Single-dimensional statistical analysis included Student’s t-test. The R software was used to draw the volcano plots.

**Fig. 4:**
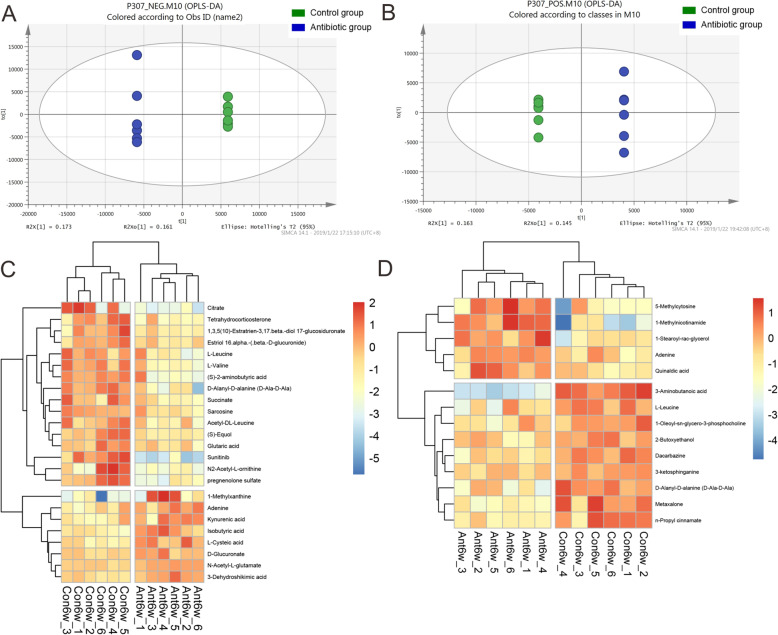
Metabolites of the gut microbiota was analyzed. **A** Orthogonal partial least squares discriminant analysis (OPLS-DA) was performed for the anionic metabolites in the two groups. **B** OPLS-DA was performed for the cationic metabolites in the two group. **C** Volcano maps constructed for the anionic metabolites in the two groups. **D** Volcano maps constructed for the cationic metabolites in the two group. (n _control group_ = 6, n _antibiotic group_ = 6)

To more comprehensively and intuitively display the relationship between samples and the levels of metabolites in different samples, we used qualitatively significant differences determined using hierarchical clustering each group of samples. We accurately screened for marker metabolites. OPLS-DA uses partial least squares regression to establish a model of the relationship between metabolite expression and sample type, which maximizes the difference between groups and reflects t1, which can directly differentiate between groupings. As shown in the Fig. 4A and B, there were obvious differences in the levels of metabolites between the groups.

The model OPLS-DA produces the VIP value; therefore, we used VIP > 1 and an independent sample t test *P* < 0.05 to screen out the differentially abundant metabolites.

Among the anionic metabolites, those showing reduced abundance in the antibiotic group compared with Control group at 6 weeks old (Ant6w/Con6w; Fig. 4C) were: L-Leucine (VIP = 5.67), Succinate (VIP = 5.19), Sunitinib (VIP = 5.05), L-Valine (VIP = 3.8), N2-Acetyl-L-ornithine (VIP = 3.44), 1,3,5(10)-Estratrien-3,17.beta.-diol17-glucosiduronate (VIP = 3.28), D-Alanyl-D-alanine (D-Ala-D-Ala) (VIP = 3.2), Citrate (VIP = 3.02), Acetyl-DL-Leucine (VIP = 2.67), Tetrahydrocorticosterone (VIP = 1.92), (S)-Equol (VIP = 1.83), pregnenolone sulfate (VIP = 1.72), Estriol 16.alpha.-(.beta.-D-glucuronide) Sarcosine (VIP = 1.47), Glutaric acid (VIP = 1.4), and (S)-2-aminobutyric acid (VIP = 1.22). The anionic metabolites showing increased abundance in the antibiotic group compared with Control group were: N-Acetyl-L-glutamate (VIP = 3.34), D-Glucuronate (VIP = 1.04), 3-Dehydroshikimic acid (VIP = 1.01), Adenine (VIP = 5.89), Kynurenic acid (VIP = 5.39), L-Cysteic acid (VIP = 1.34), Isobutyric acid (VIP = 2.18), and 1-Methylxanthine (VIP = 2.89).

Among the cationic metabolites, those showing reduced abundance in the antibiotic group compared with Control group at 6 weeks old (Ant6w/Con6w; Fig. 4D) were: 1-Oleoyl-sn-glycero-3-phosphocholin (VIP = 8.46), L-Leucine (VIP = 4.94), Metaxalone (VIP = 2.15), 3-Aminobutanoic acid (VIP = 1.93), 3-ketosphinganine (VIP = 1.88), n-Propyl cinnamate (VIP = 1.69), 2-Butoxyethanol (VIP = 1.45), D-Alanyl-D-alanine (D-Ala-D-Ala) (VIP = 1.35), and Dacarbazine (VIP = 1.05). Those showing increased abundance in the antibiotic group compared with Control group were: Adenine (VIP = 10.5), 1-Methylnicotinamide (VIP = 7.63), 5-Methylcytosine (VIP = 2.7), 1-Stearoyl-rac-glycerol (VIP = 1.81), and Quinaldic acid (VIP = 1.38).

### Analysis of the relationship between differential metabolites and gut microbiota

**Fig. 5:**
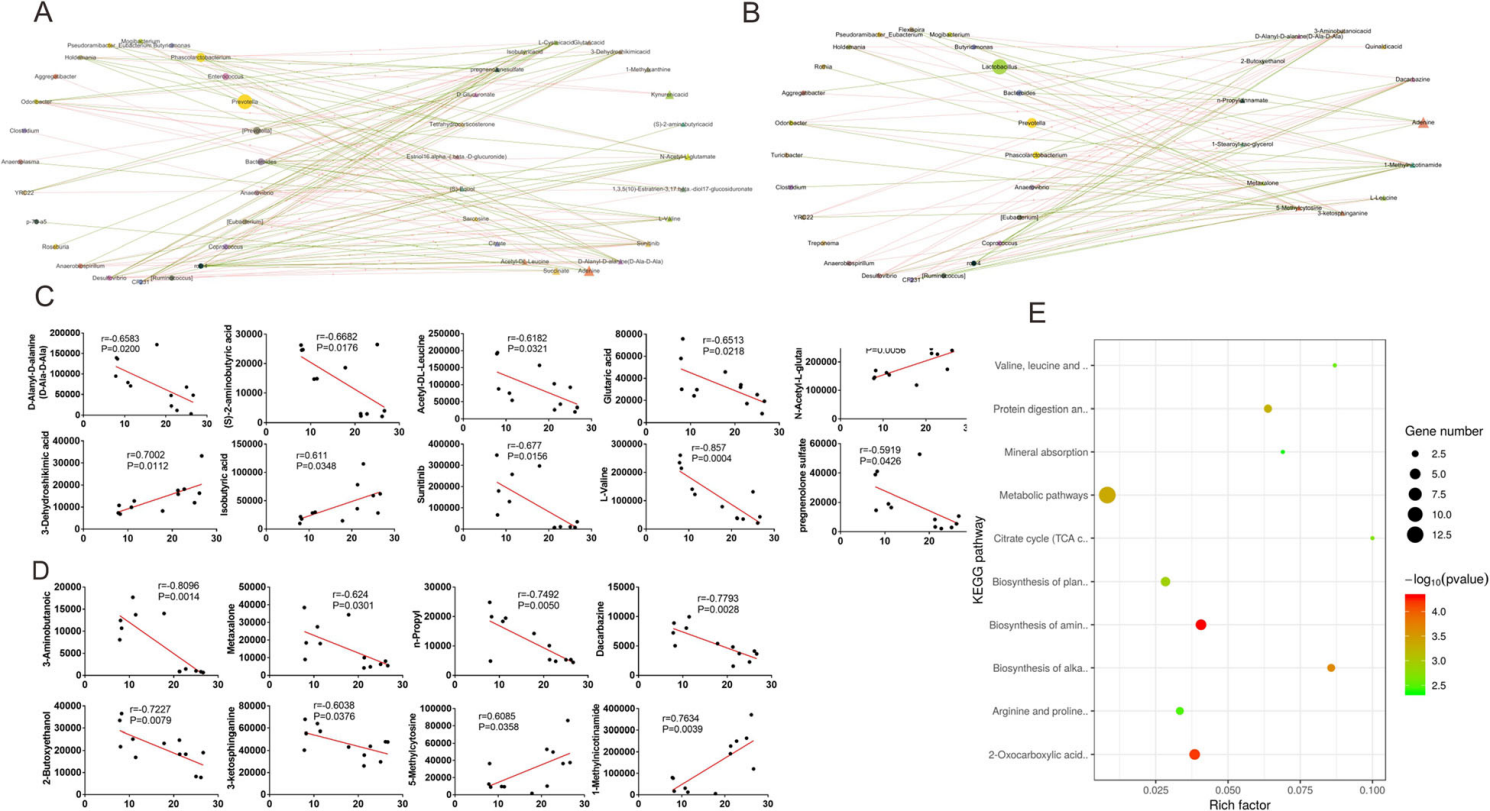
The relationship between metabolites and the gut microbiota. **A** Associated network analysis between the flora species and the anionic metabolites. Circular nodes indicate bacterial flora, triangular nodes indicate metabolomics, connections between nodes indicate correlations, in which red lines indicate positive correlations, and green lines indicate negative correlations (screening criteria: *r* > 0.6, *p* < 0.05). The more connections through a node, the more information about the flora or metabolome associated with it. The larger the area of the node, the higher the species abundance or detection index value represented by the node. **B** Associated network analysis between the flora species and the cationic metabolites. **C** Pearson correlation analysis of body weight-related unique flora and the anion metabolites. **D** Pearson correlation analysis of body weight-related unique flora and the cationic metabolites. **E** Differential metabolites Kyoto Encyclopedia of Genes and Genomes (KEGG) pathway analysis. The obtained differential metabolites (including positive and negative ion mode results) were submitted to the KEGG website for relevant pathway analysis. (n _control group_ = 6, n _antibiotic group_ = 6)

To study the relationship between differential metabolites and gut microbiota, and to determine how they affect the growth of offspring, we used Mothur software to calculate the Spearman rank correlation coefficient between the metabolome data and the microbiota abundance. We constructed a correlation network for the relevant information, using the criteria |rho| > 0.6 and the *P* value < 0.05, and imported the results into the Cytoscape software for visualization (Fig. 5A,B).

At the same time, we analyzed the correlation between differential metabolites and the abundance S24–7, which is related to body weight. The results showed that the abundance of S24–7 was related to the anionic metabolites Sunitinib, pregnenolone sulfate D-Alanyl-D-alanine (D-Ala-D-Ala), L-Valine, (S)-2-aminobutyric acid, Acetyl-DL-Leucine, Glutaric acid, N Acetyl-L-glutamate, 3-Dehydroshikimic acid, and Isobutyric acid (Fig. 5C); and the cationic metabolites 3-Aminobutanoic acid, Metaxalone, n-Propyl cinnamate, Dacarbazine, 2-Butoxyethanol, 3-ketosphinganine, 5-Methylcytosine, and 1-Methylnicotinamide (Fig. 5D).

Finally we submitted the obtained differentially abundant metabolites (including positive and negative ion model results) to the KEGG website for related pathway analysis (Fig. 5E). The pathway analysis results showed that the metabolic pathways in the feces of the antibiotic group compared with the control group changed significantly: Valine, leucine and isoleucine biosynthesis, Protein digestion and absorption, Mineral absorption, Metabolic pathways, Citrate cycle (TCA cycle), Biosynthesis of plant secondary metabolites, Biosynthesis of plant hormones, Biosynthesis of amino acids, Biosynthesis of alkaloids derived from histidine and purine, and Arginine and proline metabolism.

## Discussion

### Antibiotic exposure in pregnancy has a lasting adverse effect on weight of offspring

Due to infection, inflammation or other high-risk factors during pregnancy, the probability of antibiotic exposure is increasing year by year [30] and Ceftriaxone sodium, as an effective therapeutic drug, is widely used. This type of β-lactam antibiotic has been found to induce obvious side effects, such as immune related adverse events up to 18.4% in a recent study of adverse drug reactions in pregnant women [4], which could also affect the gut microbiota by causing changes or shifts in the number of dominant flora, resulting in an imbalance. To study the effect of Ceftriaxone sodium exposure during pregnancy on offspring, we added it to the daily drinking water of SD rats. DNA sequencing of bacteria in feces showed that the structure of gut microbiota of the pregnant rats was obviously disordered.

We assessed the weight gain changes in the offspring of the antibiotic-treated rates. The results showed no significant difference at birth; however, the rats in the antibiotic group were heavier than those in the control group at 2 weeks, but lighter at 6 weeks. These results suggested that a continuous effect of maternal antibiotic exposure on the growth of offspring does exist, and the antibiotics could directly affect the structure of the gut microbiota. Stanislawski et al. [21] found similar results. Compared with the research [1], we found no significant difference in weight at birth of offspring, which might be related to the different research object, i.e., it is likely that a different antibiotics have effects on specific flora, which might have different effects on the growth of the offspring.

### The colonization of different gut microbiota participate in weight changes of offspring

To explore whether the exposure of antibiotics during pregnancy had an impact on the gut microbiota of the offspring, we extracted the feces for bacterial DNA sequencing.

The results α diversity analysis showed no significant difference between the control group and the antibiotic exposure group; however, the β diversity analysis showed significant differences in the structure of two groups of bacteria, which was consistent with the results [7].

The results of taxonomic composition analysis showed that the dominant bacteria in the control and antibiotic groups 3 weeks old at the phylum level were mainly Firmicutes and Bacteroidetes, although their abundances varied between the groups. At 6 weeks old, the dominant bacteria in Control were Firmicutes and Bacteroidetes, and in the antibiotic group were Firmicutes, Bacteroidetes, and Chlamydiae. The predominant phyla in the gut microbiota of rats are Firmicutes and Pseudobacteria [25]. Firmicutes are believed to be related to obesity, cardiovascular disease, diabetes, and chronic intestinal inflammation. Studies have found that the proportion of Firmicutes in obese patients was significantly increased, while the proportion of Bacteroides was significantly decreased [11]. In the study of Gao et al., the abundance of Firmicutes, Bacteroidetes, and Chlamydiae were found to be related to weight change, which was consistent with our results.

At the genus level, the dominant bacteria in the Control and antibiotic groups were broadly similar but with certain differences. In particular, the analysis showed the relative abundance of S24–7 in the offspring of the antibiotic group gradually increased, which was significantly different from that of the Control group, and was associated with weight lost. Studies [31] found that S24–7, Lactobacillus, Clostridiales, and Prevotella participated in the change in body weight and confirmed that the abundance of S24–7 correlated with body weight, which is consistent with our results. In a rat model fed on a high-fat diet) [23], S24–7 and Lactobacillus correlated with weight loss. In addition, in animal models of type 2 diabetes [29] and in population studies [19], S24–7 was observed to participate in the body’s metabolism and its abundance correlated negatively with weight, which also supported our results. Therefore, we believe that gut microbiota is involved in the weight change of offspring, particularly S24–7.

### Different metabolites might affect the weight changes of offspring

To study the effects of the metabolites produced by the gut microbiota, we sampled the feces of the offspring at 6 weeks old and performed non-targeted metabolomics analysis based on UHPLC and identified differentially abundant metabolites. We then carried out correlation analysis of the differentially abundant metabolites to explore their relationships. We found that Sunitinib, Citrate, N2-Acetyl-L-ornithine, D-Alanyl-D-alanine, L-Valine, Tetrahydrocorticosterone, (S)-2-aminobutyric acid, (S)-Equol, Succinate, L-Leucine, Acetyl-DL-Leucine, Glutaric acid, Sarcosine, N-Acetyl-L-glutamate, D-Glucuronate, 3-Dehydroshikimic acid, Adenine, Kynurenic acid, L-Cysteic acid, Isobutyric acid, 1-Methylxanthine, N-Acetyl-L-glutamate, D-Glucuronate, 3-Dehydroshikimic acid, Adenine, Kynurenic acid, L-Cysteic acid, Isobutyric acid and 1-Methylxanthine correlated with body weight. Similarly, Some studies [3] also found that bacterially-produced metabolites such as valine, isoleucine, acetate, taurine, glycine, glycerol, and tryptophan, are involved in amino acid metabolism, energy metabolism, especially endocrine, lipid metabolism, and carbohydrate metabolism.

### S24–7 might affect the weight changes of offspring through different metabolic pathways

To verify whether S24–7 affects body weight through its metabolites, we performed correlation analysis and identified several anionic and cationic metabolites that were related to the abundance of S24–7. Similarly, it has been found that S24–7 is related to L-Valine, Leucine, and Glutaric acid, which are involved in the metabolism of amino acids and low cholesterol, low density lipoprotein (LDL), and fatty acid contents [22]. Sanguinetti et al. [20] also observed that S24–7 correlated negatively with L-Valine, L-Leucine, Glutaric acid, and Metaxalone, which was similar to our result.

To further identify the pathways of the metabolites related to the altered flora and that might have an effect on body weight, we submitted the differentially abundant metabolites (including positive and negative ion model results) to the KEGG website for related pathway analysis. The results suggest that antibiotic exposure during pregnancy can indeed affect the metabolic pathways of the offspring, including: Valine, leucine and isoleucine biosynthesis, Protein digestion and absorption, Mineral absorption, Metabolic pathways, and the Citrate cycle (TCA cycle). Branched chain amino acids include leucine, isoleucine and valine, which play an important role in protein synthesis and glucose homeostasis [18]. Research has confirmed that Valine, leucine, and isoleucine biosynthesis are involved in metabolic processes in humans and animals, and their lack in the diet for more than 7 days rapidly reduced the amount of abdominal fat in mice [14, 27]. Protein digestion and absorption, mineral absorption, and the citrate cycle also affect the body’s metabolism, which explains the lighter weight of the offspring exposed to antibiotics during pregnancy in our experiment, which might represent the method by which the gut microbiota to exerts biological effects via metabolites.

In summary, our study shows that antibiotic exposure in pregnancy has a long-lasting adverse effect on the growth of the offspring. After Ceftriaxone sodium exposure, the imbalance of the gut microbiota may be passed to the offspring through milk or contact, and the colonization of different gut microbiota, especially S24–7, might affect the growth of the offspring through different metabolic pathways. However, further studies are required to reveal the detailed mechanisms.

## Data Availability

The datasets generated and analysed during the current study are available in the SRA(Sequence Read Archive) of NCBI(national center for biotechnology information) repository [SRP319184].

## Abbreviations

GD: Gestational day
PND: Post-natal day
SPF: Specific Pathogen Free
UHPLC: Ultra-high performance liquid chromatography
LC-MS/MS: Liquid chromatography–tandem mass spectrometry
OPLS-DA: Orthogonal partial least squares discriminant analysis

## References

[1] Bookstaver PB, Bland CM, Griffin B, Stover KR, Eiland LS, McLaughlin M (2015). A review of antibiotic use in pregnancy. Pharmacotherapy.

[2] SW CHO, Lee JS, Choi SH (2004). Enhanced oral bio- availability of poorly absorbed drugs.I.Screening of absorption carrier for the ceftriaxone complex. J Pharm Sci.

[3] Chen R, Wang J, Zhan R, Zhang L, Wang X (2019). Fecal metabonomics combined with 16S rRNA gene sequencing to analyze the changes of gut microbiota in rats with kidney-yang deficiency syndrome and the intervention effect of you-gui pill. J Ethnopharmacol.

[4] Da Silva KDL, Fernandes FEM, de Lima PT, Lima SIVC, Oliveira AG, Martins RR (2019). Prevalence and profile of adverse drug reactions in high-risk pregnancy: a cohort study. BMC Preg Child.

[5] Dotters-Katz S (2020). Antibiotics for prophylaxis in the setting of preterm Prelabor rupture of membranes. Obstet Gynecol Clin N Am.

[6] Gonzalez-Perez G, Hicks AL, Tekieli TM, Radens CM, Williams BL, Lamousé-Smith ESN (2016). Maternal antibiotic treatment impacts development of the neonatal intestinal microbiome and antiviral immunity. J Immunol.

[7] Gao X, Jia R, Xie L, Kuang L, Feng L, Wan C (2018). A study of the correlation between obesity and intestinal flora in school-age children. Sci Rep.

[8] Heerman WJ, Daley MF, Boone-Heinonen J, Rifas-Shiman SL, Bailey LC, Forrest CB, Young JG, Gillman MW, Horgan CE, Janicke DM, Jenter C, Kharbanda EO, Lunsford D, Messito MJ, Toh S, Block JP, PCORnet. Antibiotics and Childhood Growth Study Group (2019). Maternal antibiotic use during pregnancy and childhood obesity at age 5 years. Int J Obes (Lond).

[9] Jess T, Morgen CS, Harpsøe MC, Sørensen TIA, Ajslev TA, Antvorskov JC, Allin KH (2019). Antibiotic use during pregnancy and childhood overweight: a population-based nationwide cohort study. Sci Rep.

[10] Jiang P, Trimigno A, Stanstrup J, Khakimov B, Viereck N, Engelsen SB, Sangild PT, Dragsted LO (2017). Antibiotic treatment preventing necrotising enterocolitis alters urinary and plasma metabolomes in preterm pigs. J Proteome Res.

[11] Kim MS, Bae JW (2016). Spatial disturbances in altered mucosal and luminal gut viromes of diet-induced obese mice. Environ Microbiol.

[12] Krischak MK, Rosett HA, Sachdeva S, Weaver KE, Heine RP, Denoble AE, Dotters-Katz SK (2020). Beyond expert opinion: a comparison of antibiotic regimens for infectious urinary tract pathology in pregnancy. AJP Rep.

[13] Liu Y, Du DM, Li XF (2010). Establishment of mice model for dysbiosis of intestinal flora. Chin J Microecol.

[14] Le Couteur DG, Solon-Biet SM, Cogger VC, Ribeiro R, de Cabo R, Raubenheimer D, Cooney GJ, Simpson SJ (2020). Branched chain amino acids, aging and age-related health. Ageing Res Rev.

[15] Luo X, Zheng YY, Wen RY (2016). Effects of ceftriaxone induced intestinal dysbacteriosis on lym- phocytes in different tissues in mice. Immunobiology.

[16] Mission JF, Catov J, Deihl T, Feghali M, Scifres C (2019). Antibiotic use in pregnancy, abnormal fetal growth, and development of gestational diabetes mellitus. Am J Perinatol.

[17] Mbakwa CA, Scheres L, Penders J, Mommers M, Thijs C, Arts IC (2016). Early life antibiotic exposure and weight development in children. J Pediatr.

[18] Nie C, He T, Zhang W, Zhang G, Ma X (2018). Branched chain amino acids: beyond nutrition metabolism. Int J Mol Sci.

[19] Osborne G, Wu F, Yang L, Kelly D, Hu J, Li H, Jasmine F, Kibriya MG, Parvez F, Shaheen I, Sarwar G, Ahmed A, Eunus M, Islam T, Pei Z, Ahsan H, Chen Y (2020). The association between gut microbiome and anthropometric measurements in Bangladesh. Gut Microbes.

[20] Sanguinetti E, Collado MC, Marrachelli VG (2018). Microbiome-metabolome signatures in mice genetically prone to develop dementia, fed a normal or fatty diet. Sci Rep.

[21] Stanislawski MA, Dabelea D, Wagner BD, Sontag MK, Lozupone CA, Eggesbø M (2017). Pre-pregnancy weight, gestational weight gain, and the gut microbiota of mothers and their infants. Microbiome.

[22] Seo SH, Unno T, Park SE, Kim EJ, Lee YM, Na CS, Son HS (2019). Korean traditional medicine (Jakyakgamcho-tang) ameliorates colitis by regulating gut microbiota. Metabolites.

[23] Tang W, Yao X, Xia F, Yang M, Chen Z, Zhou B, Liu Q (2018). Modulation of the gut microbiota in rats by Hugan Qingzhi tablets during the treatment of high-fat-diet-induced nonalcoholic fatty liver disease. Oxid Med Cell Longev.

[24] Vidal AC, Murphy SK, Murtha AP (2013). Associations between antibiotic exposure during pregnancy, birth weight and aberrant methylation at imprinted genes among offspring. Int J Obes.

[25] Wexler HM (2007). Bacteroides: the good, the bad, and the nitty-gritty. Clin Microbiol Rev.

[26] Wan S, Guo M, Zhang T, Chen Q, Wu M, Teng F, Long Y, Jiang Z, Xu Y (2020). Impact of exposure to antibiotics during pregnancy and infancy on childhood obesity: a systematic review and meta-analysis. Obesity (Silver Spring).

[27] Xiao F, Du Y, Lv Z (2016). Effects of essential amino acids on lipid metabolism in mice and humans. J Mol Endocrinol.

[28] Yu M, Jia HM, Zhou C, Yang Y, Sun LL, Zou ZM (2017). Urinary and fecal metabonomics study of the protective effect of chaihu-shu-gan-san on antibiotic-induced gut microbiota dysbiosis in rats. Sci Rep.

[29] Yu F, Han W, Zhan G, Li S, Jiang X, Wang L, Xiang S, Zhu B, Yang L, Luo A, Hua F, Yang C (2019). Abnormal gut microbiota composition contributes to the development of type 2 diabetes mellitus in db/db mice. Aging (Albany NY).

[30] Yoon BH, Romero R, Park JY, Oh KJ, Lee J, Conde-Agudelo A, Hong JS (2019). Antibiotic administration can eradicate intra-amniotic infection or intra-amniotic inflammation in a subset of patients with preterm labor and intact membranes. Am J Obstet Gynecol.

[31] Zhao L, Zhang Q, Ma W, Tian F, Shen H, Zhou M (2017). A combination of quercetin and resveratrol reduces obesity in high-fat diet-fed rats by modulation of gut microbiota. Food Funct.

